# Supplementary material for: Consequences of Type-2 diabetes mellitus and Malaria co-morbidity on sperm parameters in men; a case-control study in a district hospital in the Ashanti Region of Ghana
Source: PLoS One. 2023 Sep 28;18(9):e0286041. doi: 10.1371/journal.pone.0286041 (PMC10538753; doi:10.1371/journal.pone.0286041)
Supplement: S1 Table — T2DM & Malaria Co-morbidity Group = participants who had both Type-2 diabetes mellitus and malaria infection, T2DM only = participants who had only Type-2 diabetes mellitus and No T2DM & No Malaria Group = the control population, thus, participants who had neither Type-2 diabetes mellitus nor malaria infection, FBG = Fasting Blood Glucose, HbA1c-DCCT = Glycated haemoglobin. (DOCX) [file pone.0286041.s002.docx]

| **Variables** | **T2DM & Malaria Co-morbidity**  **(N=80)** | **T2DM only**  **(N=80)** | **(Control)**  **(N=94)** |
| --- | --- | --- | --- |
| **FBG (mmol/L)** | 11.82±4.45 | 11.33±2.92 | 4.84±.53 |
| **HbA1c-DCCT (%)** | 11.13±3.94 | 10.46±2.62 | 5.18±.32 |
| **Testosterone (ng/mL)** | 4.13±.95 | 5.62±.70 | 7.32±.89 |
