# Supplementary material for: Consequences of Type-2 diabetes mellitus and Malaria co-morbidity on sperm parameters in men; a case-control study in a district hospital in the Ashanti Region of Ghana
Source: PLoS One. 2023 Sep 28;18(9):e0286041. doi: 10.1371/journal.pone.0286041 (PMC10538753; doi:10.1371/journal.pone.0286041)
Supplement: S2 Table — T2DM & Malaria Co-morbidity Group = participants who had both Type-2 diabetes mellitus and malaria infection, T2DM only = participants who had only Type-2 diabetes mellitus and No T2DM & No Malaria Group = the control population, thus, participants who had neither Type-2 diabetes mellitus nor malaria infection. (DOCX) [file pone.0286041.s003.docx]

| **Variables** | **T2DM & Malaria Co-morbidity**  **(N=80)** | **T2DM only**  **(N=80)** | **(Control)**  **(N=94)** |
| --- | --- | --- | --- |
| **Volume of semen** | 2.34±.54 | 2.37±.36 | 2.78±.69 |
| **Total motility (A+B) (%)** | 35.43±11.91 | 47.98±9.57 | 75.64±6.31 |
| **Rapid progressive motility A %** | 19.30±8.39 | 26.38±6.41 | 46.85±5.39 |
| **Slow progressive motility B %** | 16.25±5.86 | 21.85±4.71 | 28.79±3.44 |
| **Non progressive motility (C) %** | 7.05±4.44 | 6.4250±4.82 | 10.28±5.07 |
| **Immotile sperm (D)** | 57.53±13.37 | 45.60±10.90 | 14.19±6.49 |
| **Sperm concentration (x10^6/mL** | 13.51±9.91 | 18.14±4.47 | 56.98±15.39 |
| **Total sperm countx10^6/ejaculate** | 31.81±26.73 | 43.53±15.56 | 159.76±59.50 |
| **Morphology normal forms (%)** | 45.78±15.65 | 57.18±6.34 | 73.30±6.70 |
| **Morphology abnormal forms %** | 54.10±15.67 | 42.83±6.34 | 26.70±6.70 |
