# Supplementary material for: Consequences of Type-2 diabetes mellitus and Malaria co-morbidity on sperm parameters in men; a case-control study in a district hospital in the Ashanti Region of Ghana
Source: PLoS One. 2023 Sep 28;18(9):e0286041. doi: 10.1371/journal.pone.0286041 (PMC10538753; doi:10.1371/journal.pone.0286041)
Supplement: S4 Table — T2DM & Malaria Co-morbidity Group = participants who had both Type-2 diabetes mellitus and malaria infection, T2DM only = participants who had only Type-2 diabetes mellitus; Data represented Mean±SD. Mean difference was significant at α<0.05. * Statistically significant difference between T2DM & Malaria Co-morbidity Group and T2DM Only. (DOCX) [file pone.0286041.s005.docx]

| **Variables** | **T2DM & Malaria Co-morbidity (N=80)** | **T2DM only**  **(N=80)** | **P-value** |
| --- | --- | --- | --- |
| **Fasting blood glucose (mmol/L)** | 11.82±4.45 | 11.33±2.92 | 0.828 |
| **HbA1c-DCCT (%)** | 11.13±3.94 | 10.46±2.62 | 0.642 |
| **Testosterone (ng/mL)** | 4.13±.95 | 5.62±.70 | ˂0.0001* |
| **Volume of semen** | 2.34±.54 | 2.37±.36 | 0.946 |
| **Total motility (A+B) (%)** | 35.43±11.91 | 47.98±9.57 | ˂0.0001* |
| **Rapid progressive motility A %** | 19.30±8.39 | 26.38±6.41 | ˂0.0001* |
| **Slow progressive motility B %** | 16.25±5.86 | 21.85±4.71 | ˂0.0001* |
| **Non progressive motility (C) %** | 7.05±4.44 | 6.4250±4.82 | 0.819 |
| **Immotile sperm (D)** | 57.53±13.37 | 45.60±10.90 | ˂0.0001* |
| **Sperm concentration (x10^6/mL** | 13.51±9.91 | 18.14±4.47 | 0.157 |
| **Total sperm countx10^6/ejaculate** | 31.81±26.73 | 43.53±15.56 | 0.05 |
| **Morphology normal forms (%)** | 45.78±15.65 | 57.18±6.34 | ˂0.0001* |
| **Morphology abnormal forms %** | 54.10±15.67 | 42.83±6.34 | ˂0.0001* |
