# Supplementary material for: Consequences of Type-2 diabetes mellitus and Malaria co-morbidity on sperm parameters in men; a case-control study in a district hospital in the Ashanti Region of Ghana
Source: PLoS One. 2023 Sep 28;18(9):e0286041. doi: 10.1371/journal.pone.0286041 (PMC10538753; doi:10.1371/journal.pone.0286041)
Supplement: S5 Table — T2DM & Malaria Co-morbidity Group = participants who had both Type-2 diabetes mellitus and malaria infection and No T2DM & No Malaria Group = the control population. Data represented Mean±SD. Mean difference was significant at α<0.05. * Statistically significant difference between T2DM & Malaria Co-morbidity Group and Control Group. (DOCX) [file pone.0286041.s006.docx]

| **Variables** | **T2DM & Malaria Co-morbidity (N=80)** | **(Control)**  **(N=94)** | **P-value** |
| --- | --- | --- | --- |
| **Fasting blood glucose (mmol/L)** | 11.82±4.45 | 4.84±.53 | ˂0.0001* |
| **HbA1c-DCCT (%)** | 11.13±3.94 | 5.18±.32 | ˂0.0001* |
| **Testosterone (ng/mL)** | 4.13±.95 | 7.32±.89 | ˂0.0001* |
| **Volume of semen** | 2.34±.54 | 2.78±.69 | 0.001* |
| **Total motility (A+B) (%)** | 35.43±11.91 | 75.64±6.31 | ˂0.0001* |
| **Rapid progressive motility A %** | 19.30±8.39 | 46.85±5.39 | ˂0.0001* |
| **Slow progressive motility B %** | 16.25±5.86 | 28.79±3.44 | ˂0.0001* |
| **Non progressive motility (C) %** | 7.05±4.44 | 10.28±5.07 | 0.006* |
| **Immotile sperm (D)** | 57.53±13.37 | 14.19±6.49 | ˂0.0001* |
| **Sperm concentration (x10^6/mL** | 13.51±9.91 | 56.98±15.39 | ˂0.0001* |
| **Total sperm countx10^6/ejaculate** | 31.81±26.73 | 159.76±59.50 | ˂0.0001* |
| **Morphology normal forms (%)** | 45.78±15.65 | 73.30±6.70 | ˂0.0001* |
| **Morphology abnormal forms %** | 54.10±15.67 | 26.70±6.70 | ˂0.0001* |
