# Supplementary material for: Consequences of Type-2 diabetes mellitus and Malaria co-morbidity on sperm parameters in men; a case-control study in a district hospital in the Ashanti Region of Ghana
Source: PLoS One. 2023 Sep 28;18(9):e0286041. doi: 10.1371/journal.pone.0286041 (PMC10538753; doi:10.1371/journal.pone.0286041)
Supplement: S6 Table — T2DM only = participants who had only Type-2 diabetes mellitus and No T2DM & No Malaria Group = the control population. Data represented Mean±SD. Mean difference was significant at α<0.05. * Statistically significant difference between T2DM Only and the Control Group. (DOCX) [file pone.0286041.s007.docx]

| **Variables** | **T2DM only**  **(N=80)** | **(Control)**  **(N=94)** | **P-value** |
| --- | --- | --- | --- |
| **Fasting blood glucose (mmol/L)** | 11.33±2.92 | 4.84±.53 | ˂0.0001* |
| **HbA1c-DCCT (%)** | 10.46±2.62 | 5.18±.32 | ˂0.0001* |
| **Testosterone (ng/mL)** | 5.62±.70 | 7.32±.89 | ˂0.0001* |
| **Volume of semen** | 2.37±.36 | 2.78±.69 | 0.002* |
| **Total motility (A+B) (%)** | 47.98±9.57 | 75.64±6.31 | ˂0.0001* |
| **Rapid progressive motility A %** | 26.38±6.41 | 46.85±5.39 | ˂0.0001* |
| **Slow progressive motility B %** | 21.85±4.71 | 28.79±3.44 | ˂0.0001* |
| **Non progressive motility (C) %** | 6.4250±4.82 | 10.28±5.07 | 0.0008* |
| **Immotile sperm (D)** | 45.60±10.90 | 14.19±6.49 | ˂0.0001* |
| **Sperm concentration (x10^6/mL** | 18.14±4.47 | 56.98±15.39 | ˂0.0001* |
| **Total sperm countx10^6/ejaculate** | 43.53±15.56 | 159.76±59.50 | ˂0.0001* |
| **Morphology normal forms (%)** | 57.18±6.34 | 73.30±6.70 | ˂0.0001* |
| **Morphology abnormal forms %** | 42.83±6.34 | 26.70±6.70 | ˂0.0001* |
