# Supplementary material for: Consequences of Type-2 diabetes mellitus and Malaria co-morbidity on sperm parameters in men; a case-control study in a district hospital in the Ashanti Region of Ghana
Source: PLoS One. 2023 Sep 28;18(9):e0286041. doi: 10.1371/journal.pone.0286041 (PMC10538753; doi:10.1371/journal.pone.0286041)
Supplement: Attachment — Submitted filename: Response to Reviewers.docx [file pone.0286041.s008.docx]

**Point-by-Point Responses to Reviewers’ Comments**

Date: 01/05/2023

Submission ID: PONE-D-23-06530

Title of Article: Consequences of type-2 diabetes mellitus and malaria co-morbidity on sperm parameters in men; a case-control study in a district hospital in the Ashanti Region of Ghana.

Name of the Corresponding Author: Benjamin Aboagye

Email Address of the Corresponding Author: [baboagye@ucc.edu.gh](mailto:baboagye@ucc.edu.gh)

Dear Editors and Reviewers,

We greatly appreciate the thorough and thoughtful comments provided on our submitted manuscript. We have addressed and revised accordingly all the comments of the reviewer(s). Attached are our detailed responses to their comments. All changes/clarifications in the manuscript have been highlighted in yellow.

We appreciate your consideration of this manuscript. Please find enclosed our revised manuscript.

Sincerely,

The Authors of the Manuscript: PONE-D-23-06530

**POINT-BY-POINT RESPONSES TO REVIEWERS**

Reviewer #1

The paper is quite good but there are some typographical and grammatical errors, the commonest of which is putting periods after headings.

Authors Response: We have tried to provide clarification by addressing the issues raised below:

There should be a description of the hospital (is it in an urban or rural area? How many bedded hospital is it? Does it have a diabetes clinic?

Authors Response: The description of the Effiduase Government Hospital has been made in the manuscript. The hospital is a district hospital with 150 bed capacity and also has a diabetic clinic. (Page 3, lines 72-79)

The type of testosterone assay was not well established under the methodology as the authors did not state if it was free or total or bioavailable testosterone that was measured. Was there any other hormone assay (prolactin, estradiol performed?).

Authors Response: Total testosterone was the type of testosterone measured. No other hormonal assay was done because the study only sort to investigate the influence of T2DM and Malaria co-morbidity on testosterone secretion since it plays a direct role in spermatogenesis, sperm maturation and overall sperm health. (Page 7, line 130, lines 134-136)

Also the references need to be well standardised and seem not to follow any certain citing style/pattern especially those from online (internet) journals.

Authors Response: The references have been standardised and cited proper in the manuscript. (Page 23-29)

Reviewer #2

Study reports a very important aspect globally; and mostly in Africa where infertility carries important medical and mostly psychosocial implications. The analysis of outcomes has used standard methods, for both malaria and diabetes; results are very relevant and matched with the research questions. The design is appropriate, and the different essays have been conducted following the standardized methods.

1. However, the application of the design presents some weaknesses which introduced doubt in the findings concerning introduction of possible bias.

Authors Response: Case-control studies just like other study designs have some limitations, a chief of these is recall bias. However, the inferential statistics was conducted using measured parameters. The possible limitations that may have presented weaknesses in the findings have been addressed in the limitation of the study. (Page 22, lines 395-401)

2. We suggest reworking on following observations and revised all criteria of a good case-control study to increase even more the quality of this study. The design of the study is quite good, and the conduct of the study was smoothly conducted. The technical aspects related to equipment’s lab techniques used by the author’s team was appreciable; however, the case-control aspect has been a bit neglected. Several aspects reported in case-control studies were not mentioned here (see methodology and even an aspect of the results) Using the checklist of reporting good case-control studies, we can realize for example:

- Assignment of patients in groups of the study?

Authors Response: Assignment of study participants in groups has been addressed in the study participants section. “The study participants comprised 254 consented adult males, 160 of them had registered and were receiving treatment at the diabetic clinic of the Effiduase Government Hospital and 94 control participants comprised of healthy blood donors, members of staff of EDH, and those visiting their relatives on hospital admission. Out of the 160 Type-2 diabetic men, 80 of them had only T2DM and the other 80 had T2DM co-morbid with malaria infection”. (Page 4, lines 81-85)

- Analysis not detailed, stratification-logistic regression, which assumptions?

Authors Response: The aim of this study sort to compare of means of the various sperm parameters within the three groups (T2DM only, T2DM & Malaria co-morbidity and the Control or the Healthy groups), hence the application of a One-way ANOVA and subsequently Tukey’s HSD post hoc tests for multiple comparisons of means of measured indices and not stratification-logistic regression.

- Socio-demographic data is not presented stratified by groups (case/controls) to get an idea about similarities/differences between the two groups

Authors Response: We have now presented the socio-demographic data in a stratified manner by groups namely; T2DM only, T2DM & Malaria co-morbidity and the Control or the Healthy groups).

Editor’s comment

While revising your submission, please upload your figure files to the Preflight Analysis and Conversion Engine (PACE) digital diagnostic tool, https://pacev2.apexcovantage.com/. PACE helps ensure that figures meet PLOS requirements. To use PACE, you must first register as a user. Registration is free. Then, login and navigate to the UPLOAD tab, where you will find detailed instructions on how to use the tool. If you encounter any issues or have any questions when using PACE, please email PLOS at figures@plos.org. Please note that Supporting Information files do not need this step.

**Authors Response:** Figure files are now in PACE format.

**Editor’s comment:** Please clarify the Tables' numbers uploaded in your PDF file.

**Authors Response:** The tables are numbered from “Table 1-4” and each table is found right underneath their description.
